# Drug-Induced Liver Injury From Sacubitril-Valsartan Versus a Single Dose of Acarbose

**DOI:** 10.7759/cureus.27005

**Published:** 2022-07-19

**Authors:** Supraja Achuthanandan, Amit Dhaliwal, Ravikaran Patti

**Affiliations:** 1 Internal Medicine, Maimonides Medical Center, Brooklyn, USA

**Keywords:** acute liver failure (alf), drug-induced acute liver failure, acarbose, sacubitril-valsartan, entrestro, drug-induced liver injury (dili)

## Abstract

Numerous known medications can induce liver injury. Sacubitril-valsartan was approved by the Food and Drug Administration in 2015 for use in patients with chronic heart failure to reduce the rate of cardiovascular death and hospitalizations related to heart failure. There are yet to be any reported cases of drug-induced liver injury secondary to sacubitril-valsartan use. Acarbose is well known to be associated with liver failure, but typically liver injury occurs months after initiation of therapy. Here, we report the case of a 76-year-old woman who developed acute liver failure after one month of sacubitril-valsartan use and one day after being prescribed acarbose.

## Introduction

Drug-induced liver injury (DILI) is the leading cause of acute liver failure [1]. Clinical presentation can vary, mimicking either acute or chronic liver injury; with over 1,000 medications and supplements implicated in causing DILI, a high index of clinical suspicion is needed to make a diagnosis. Oftentimes the diagnosis is supported when the removal of the offending agents results in rapid improvement of the patient’s liver function tests. Acarbose is an alpha-glucosidase inhibitor and has a known dose-dependent hepatotoxic effect occurring months after exposure to the drug [2]. Valsartan, an angiotensin-receptor blocking agent, has been rarely reported to cause DILI [3]. Sacubitril, a neprilysin inhibitor approved for use in combination with valsartan in patients with congestive heart failure, has not been noted to have any reported cases of DILI. Here, we report the case of an elderly woman who was started on acarbose one day prior to presentation and sacubitril-valsartan one month prior to presentation and was found to have DILI.

## Case presentation

A 76-year-old woman with a history of diabetes mellitus, hypertension, hyperlipidemia, congestive heart failure with an ejection fraction of 36-40%, and heart block status post-pacemaker presented to the hospital for increasing lethargy for four days. She had become increasingly somnolent which prompted her family to bring her to the hospital. She denied any fevers, chills, chest pain, shortness of breath, nausea or vomiting, abdominal pain, diarrhea, or urinary complaints. She denied any sick contacts or recent travel. She had never smoked and denied the use of herbal supplements, alcohol, or illicit drugs. She did not have a family history of liver disease. The patient was of Indian descent; she was born in India and had been living in the United States for the last 30 years.

The patient was previously insulin dependent; however, her recent bloodwork from one week prior revealed improvement in her HbA1c, prompting her primary care doctor to discontinue it. Since discontinuation, her home blood glucose measurements were over 300 mg/dL, for which she was prescribed acarbose one day prior to presentation. The patient had only taken one dose before the presentation. The only other new medication was sacubitril-valsartan, which was started one month prior. Her full medication list was as follows: acarbose, sacubitril-valsartan, furosemide, metoprolol succinate, pravastatin, metformin, magnesium oxide, and as needed acetaminophen-dextromethorphan-doxylamine (which she had taken only twice in the one week prior to her hospitalization).

On examination, the patient was afebrile, hemodynamically stable, and breathing comfortably on room air. She was alert and oriented to self and place but not to time. No focal neurologic deficits were elicited. No jaundice, tremors, or asterixis was noted. Cardiac and lung examinations were within normal limits. Her abdomen was soft, non-distended, and non-tender. There was no right upper quadrant tenderness or palpable hepatomegaly. Moreover, there was no jugular venous distention or lower extremity edema.

Significant laboratory findings were as follows: white blood cell count 15.8 K/UL, hemoglobin 11.4 g/dL, platelets 239 K/UL, sodium 129 mmol/L, potassium 6.2 mmol/L, bicarbonate 13 mmol/L, blood urea nitrogen 59 mg/dL, creatinine 2.2 mg/dL, lactic acid 12 mmol/L, and anion gap 27. Her liver function tests (LFTs) revealed albumin 3.5 g/dL, total bilirubin 1.2 mg/dL, direct bilirubin 0.3 mg/dL, aspartate aminotransferase (AST) 1,578 IU/L, alanine aminotransferase (ALT) 1,652 IU/L, alkaline phosphatase (ALP) 55 IU/L, gamma-glutamyl transferase (GGT) 23 IU/L, and ammonia 85 µmol/L. Of note, the bloodwork performed one week prior had revealed normal electrolytes and LFTs. Her coagulation profile showed a prothrombin time of 32.8 seconds and an international normalized ratio of 2.8. Serum acetaminophen level was <10 µg/mL, and a hepatitis panel was non-reactive. Finally, serology was negative for antinuclear antibody, anti-smooth muscle antibody, alpha-1 antitrypsin antibody, and anti-liver-kidney microsomal antibody.

**Figure 1:**
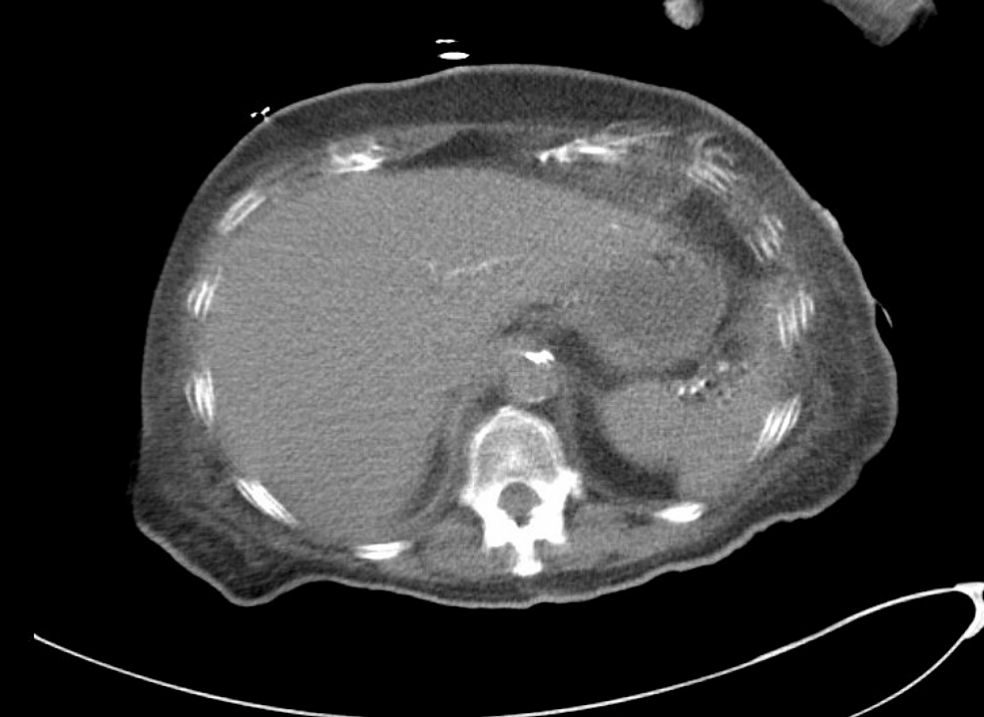
Computed tomography without contrast. The liver is noted to be within normal limits in regards to size and contour, without the presence of any focal masses.

**Figure 2:**
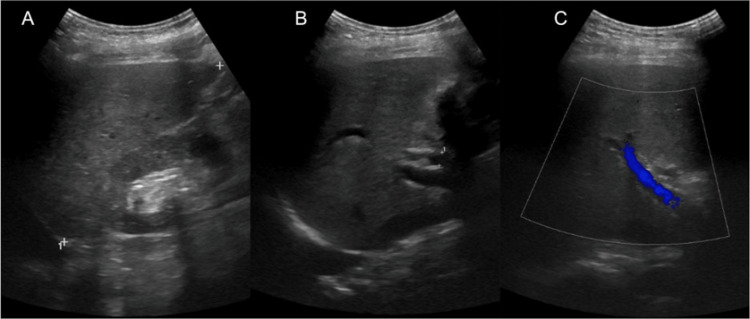
Ultrasound of the liver. (A) Echogenic liver with normal contour, measuring 12 cm. (B) The common bile duct measuring 7 mm. (C) A patent portal vein.

Chest X-ray and computed tomography (CT) of the head revealed no acute pathologies. CT of the abdomen and pelvis without intravenous contrast showed atrophic kidneys, cholelithiasis, and a liver which appeared normal in size and contour (Figure 1). Liver ultrasound revealed coarse echogenicity of the liver with normal size and contour, no visible masses, and a patent portal vein with a normal direction of portal vein flow (Figure 2).

The patient was admitted to the intensive care unit for management of elevated anion gap metabolic acidosis secondary to lactic acidosis, suspected DILI with acute liver failure, and acute kidney injury. Her Child-Pugh score was Class B and her Na-Model for End-Stage Liver Disease (MELD) score was 30 (indicating three-month mortality of 52.6%). She was seen by the gastroenterology team for a possible liver transplant; however, due to her age and comorbidities, she was not a candidate. She was started on N-acetylcysteine infusion in the setting of acute liver failure, and rifaximin and lactulose for hepatic encephalopathy. The patient had significant improvement in her laboratory abnormalities and clinical status throughout her hospital stay and she was discharged on day eight. She was thought to have DILI secondary to either acarbose or sacubitril-valsartan, thus both medications were discontinued, and she was discharged instead on losartan and insulin. Her LFTs were repeated two weeks after discharge and were normal, thus pravastatin was resumed. LFTs were again repeated four months after discharge and remained normal.

## Discussion

DILI can be caused by several medications and herbal supplements. Approximately 1 in 10,000 to 1 in 100,000 cases of liver injury are reported to be caused by medications [4]. However, DILI accounts for up to 10% of acute hepatitis and is the leading cause of acute liver failure [1]. In the United States, the most common drugs to known cause DILI include acetaminophen, antibiotics, and antiepileptics [5]. Clinical presentation varies as it can mimic any form of liver disease, acute or chronic, making it a difficult diagnosis. Clinical presentation is based on the type of hepatic injury, namely, hepatocellular pattern, cholestatic pattern, or mixed pattern [1,4]. Most patients are actually asymptomatic but can present with abdominal pain and weakness, or in the case of cholestatic liver injury, can have jaundice and dark urine [1]. DILI is a diagnosis of exclusion, and other causes of cytotoxic/cholestatic liver injury must be excluded such as viral hepatitis, ischemic hepatitis, congestive hepatopathy, non-alcoholic fatty liver disease, cholangitis, and choledocholithiasis [6,7].

The diagnosis of DILI involves maintaining a high index of suspicion in the right patient with recent changes in medications that have been implicated to cause DILI. Diagnosis can be made when at least one of the following are met: elevation of ALT ≥5× the upper limit of normal (ULN), or ALT ≥3× ULN with total bilirubin >2× ULN, or ALP ≥2× ULN [8]. The type of DILI can be further classified as hepatocellular, cholestatic, or mixed. A hepatocellular liver injury is diagnosed when ALT ≥5× ULN or if ALT/ALP ≥5. A cholestatic pattern is diagnosed when ALP ≥2× ULN or if ALT/ALP ≤2. Lastly, a mixed pattern is suspected in ALT/ALP is 2-5 [8]. Both the diagnosis and treatment of DILI involve the removal of offending agent(s) with rapid improvement in LFTs [1].

We report a case of an elderly woman who was started on acarbose one day prior to presentation and sacubitril-valsartan one month prior. The patient presented with lethargy and was found to have deranged LFTs (with normal LFTs one week prior to presentation). The patient’s ALT was elevated >5× ULN, without elevation in ALP or total bilirubin, suggesting hepatocellular injury. A myriad of tests was performed to rule out other etiologies of acute liver injury. Because these tests were negative, there was a high suspicion of DILI. The patient’s hepatotoxic medications were held, and there was rapid improvement in her LFTs which confirmed the diagnosis.

The patient was on several medications that could have caused DILI. Statins have a well-known association with hepatotoxicity and the patient’s pravastatin was held on admission. The patient had been taking pravastatin for more than 20 years. Studies have shown that hepatotoxicity associated with statins typically has a latency to onset between 34 days and 10 years [9]. Thus, it is unlikely that the patient had acute liver failure from pravastatin. The other offending agents could have been acarbose and sacubitril-valsartan, which were also held on admission. Several large clinical trials have reported dose-dependent hepatotoxicity in patients started on acarbose [2,10,11]. Our patient had taken only one dose of acarbose, one day prior to presentation, and presented with acute liver failure, which makes this a very unusual case in reference to the prior meta-analyses, which reported the development of hepatotoxicity after 2-12 months of acarbose therapy [2,11]. To our knowledge, there are no reports of hepatotoxicity from only a single dose of acarbose. Furthermore, the patient had been feeling unwell and lethargic prior to the initiation of acarbose; thus, it is unclear if acarbose alone was the culprit of DILI in this patient. The only other medication that was new for the patient was sacubitril-valsartan, which had been prescribed by the patient’s cardiologist one month prior to presentation. When reviewing the current literature, there is no evidence of either sacubitril or sacubitril-valsartan causing hepatocellular injury. However, there are sparse case reports of valsartan-induced hepatotoxicity [3]. It is possible that the combination of sacubitril-valsartan and acarbose caused liver injury in this patient.

The patient had a rapid recovery in her LFTs. DILI has a reported recovery rate of 90% [7]. Despite low evidence of single-dose acarbose-induced liver injury or sacubitril-valsartan-induced liver injury, removal of both agents resulted in rapid improvement of LFTs and mental status. She was discharged on losartan instead of sacubitril-valsartan due to reduced ejection fraction heart failure. Of note, there have been cases of losartan-induced hepatotoxicity as well, and this patient was monitored very closely as an outpatient [12]. Her LFTs were repeated two weeks and four months after discharge and remained normal. Further studies are needed to compare the incidence of DILI in patients using sacubitril-valsartan alone and in combination with acarbose.

## Conclusions

DILI is the leading cause of acute liver failure. When suspecting DILI, a detailed review of medications is crucial because uncommon drugs such as sacubitril-valsartan can be the culprit. When starting patients on acarbose, clinicians must be aware of the risk of liver injury, even with a single dose.
